# Supplementary material for: The enteric nervous system of the C. elegans pharynx is specified by the Sine oculis-like homeobox gene ceh-34
Source: eLife. 2022 Mar 24;11:e76003. doi: 10.7554/eLife.76003 (PMC8989417; doi:10.7554/eLife.76003)
Supplement: Supplementary file 1. — This file provides a list of all Caenorhabditis elegans strains used in this study. [file elife-76003-supp1.docx]

**Supplementary File 1 - Strain list**

| **Strain Name** | ***Mutant or knock-in*** | **Array name** | **DNA on array** | **Reference** |
| --- | --- | --- | --- | --- |
| OH15151 | *ceh-34(tm3733)* | *otEx7040* | *ceh-34Fosmid, myo-3prom::mCherry* | Fosmid rescue of allele described in (Amin et al., 2009) |
| OH16335 | *ceh-34(ot1014)* | *otEx7476* | *ceh-34Fosmid, myo-3prom::mCherry* | This study |
| OH17564 | *ceh-34(ot1188) flr-2(syb4861)/+ flr-2(syb4861)* |  |  | This study |
| OH17565 | *ceh-34(ot1189) htrl-1(syb4895)/+ htrl-1(syb4895* |  |  | This study |
| OH15439 | *ceh-34(ot903[ceh-34::mNG::3xFLAG::AID]* |  |  | This study |
| MT15695 | *ceh-34(n4796)* |  |  | (Hirose et al., 2010) |
| OH17817 | *eya-1(ot1197)* | *otIs661* | *unc-17prom::GFP, unc-122prom::GFP* | This study |
| SA146 | *eya-1(ok654)* |  |  | (Furuya et al., 2005) |
| MT1859 | *unc-86(n846)* |  |  | (Baumeister et al., 1996) |
| OH15422 | *ceh-14(ot900)* |  |  | (Bayer and Hobert, 2018) |
| TB200 | *ceh-2(ch4)* |  |  | (Aspock et al., 2003) |
| OH16563 | *ceh-45(ot1065)* |  |  | This study |
| OH17142 | *vab-15(ot1136); unc-17(ot907[unc-17::mKate2::3xFLAG])* |  |  | This study |
| OH17144 | *ceh-7(ot907); unc-17(ot907[unc-17::mKate2::3xFLAG])* |  |  | This study |
| OH16564 | *ceh-53(ot1066)* |  |  | This study |
| OH16565 | *ceh-79(ot1067)* |  |  | This study |
| JK1521 | *fog-2(q71) pha-4(q490)/stu-3(q265) rol-9(sc148)* |  |  | (Mango et al., 1994) |
| CX5000 | *slt-1(eh15)* |  |  | (Hao et al., 2001) |
| PHX4513 | *flp-5(syb4513 [flp-5::SL2::GFP::H2B])* |  |  | This study |
| PHX3207 | *flp-28(syb3207 [flp-28::T2A::3×NLS::GFP])* |  |  | This study |
| PHX4861 | *flr-2(syb4861 [flr-2::SL2::GFP::H2B])* |  |  | This study |
| PHX4895 | *htrl-1(syb4895 [htrl-1::SL2::GFP::H2B])* |  |  | This study |
| PHX4677 | *kin-36(syb4677[GFP::HIS::SL2::kin-36])* |  |  | This study |
| OH15876 | *pha-4(ot946 [pha-4::GFP])* |  |  | This study |
| PHX4763 | *rig-3(syb4763 [rig-3::SL2::GFP::H2B])* |  |  | This study |
| PHX4729 | *rig-6(syb4729 [rig-6::SL2::GFP::H2B])* |  |  | This study |
| PHX4502 | *ser-7(syb4502 [ser-7::SL2::GFP::H2B])* |  |  | This study |
| PHX4421 | *trh-1(syb4421 [trh-1::SL2::GFP::H2B])* |  |  | This study |
| PHX4453 | *trhr-1(syb4453 [trhr-1::SL2::GFP::H2B])* |  |  | This study |
| PHX4491 | *unc-17(syb4491[unc-17::T2A::GFP:H2B])* |  |  | This study |
| PHX4257 | *eat-4(syb4257)[eat-4::T2A::GFP::H2B])* |  |  | This study |
| OP575 | *unc-119(tm4063)* | *wgIs575* | *ceh-33fosmid::TY1::EGFP::3xFLAG, unc-119(+)* | (Sarov et al., 2012) |
| OP524 | *unc-119(tm4063)* | *wgIs524* | *ceh-34fosmid::TY1::EGFP::3xFLAG, unc-119(+)* | (Sarov et al., 2012) |
| SD1345 |  | *stIs10077* | *pha-4prom::his-24::mCherry, unc-119(+)* | (Liu et al., 2009) |
| HML1012 |  | *cshIs140* | *rps-28p::TIR1(F79G)::T2A::mCherry::his-11 + Cbr-unc-119(+)* | PMID: 34739048 |
| OH8251 |  | *otIs226* | *bas-1prom::gfp* | (Flames and Hobert, 2009) |
| OH8246 |  | *otIs221* | *cat-1prom::gfp* | (Flames and Hobert, 2009) |
| OH8259 |  | *otIs224* | *cat-1prom::gfp* | (Flames and Hobert, 2009) |
| OH8250 |  | *otIs225* | *cat-4prom::gfp* | (Flames and Hobert, 2009) |
| OH12262 |  | *otIs487* | *eat-4prom7::gfp, ttx-3prom::mCherry* | (Serrano-Saiz et al., 2020) |
| OH12797 |  | *otIs558* | *eat-4prom14::gfp, ttx-3prom::mCherry* | (Serrano-Saiz et al., 2020) |
| OH12496 |  | *otIs518* | *eat-4fosmid::SL2::mCherry, pha-1(+)* | (Serrano-Saiz et al., 2013) |
| OH11124 |  | *otIs388* | *eat-4fosmid::SL2::YFP::H2B, pha-1(+)* | (Serrano-Saiz et al., 2013) |
| MT19075 |  | *nIs352* | *eya-1p::GFP::eya-1* | (Furuya et al., 2005; Hirose et al., 2010) |
| NY2057 | *him-5(e1490)* | *ynIs57* | *flp-2prom::GFP* | (Kim and Li, 2004) |
| NY2030 |  | *ynIs30* | *flp-4prom::GFP* | (Kim and Li, 2004) |
| NY2045 | *him-5(e1490)* | *ynIs45* | *flp-15prom::GFP* | (Kim and Li, 2004) |
| NY2080 |  | *ynIs80* | *flp-21prom::GFP* | (Kim and Li, 2004) |
| OH16816 |  | *otIs809* | *glr-7::GFP, lin-15(+)* | Integrant of Ex array described in (Brockie et al., 2001) |
| MT24110 | *lin-15(n765)* | *nIs780* | *gur-3prom::GFP, lin-15(+)* | (Sando et al., 2021) |
| OH10545 |  | *otIs341* | *mgl-1prom::GFP, pha-1(+)* | (Zhang et al., 2014) |
| OH15487 |  | *otIs695* | *nlp-3prom::GFP, lin-15(+)* | Integrant of Ex array described in (Nathoo et al., 2001) |
| OH15655 |  | *otIs711* | *nlp-8prom::GFP, lin-15(+)* | Integrant of Ex array described in (Nathoo et al., 2001) |
| OH16003 |  | *otIs742* | *nlp-13prom::GFP, lin-15(+)* | Integrant of Ex array described in (Nathoo et al., 2001) |
| OH9609 |  | *otIs291* | *rab-3prom::2xNLS::YFP, rol-6(su1006)* | (Stefanakis et al., 2015) |
| OH10689 |  | *otIs355* | *rab-3prom::2xNLS::TagRFP* | (Stefanakis et al., 2015) |
| OH10684 |  | *otIs350* | *ric-4fosmid::SL2::NLS::YFP::H2B, pha-1(+)* | (Stefanakis et al., 2015) |
| OH11061 |  | *otIs380* | *ric-19prom::2xNLS::GFP, elt-2::DsRED* | (Stefanakis et al., 2015) |
| OH13606 |  | *otIs620* | *unc-11prom::2xNLS::GFP* | (Leyva-Diaz et al., 2017) |
| SD1614 |  | *stIs10447* | *ceh-34prom::HIS-24::mCherry, unc-119(+)* | (Liu et al., 2009) |
| CX4384 |  | *kyIs174* | *slt-1prom::GFP* | (Hao et al., 2001) |
| OH17563 |  | *otIs868* | *spp-12prom::spp-12(1-97)::GFP* | Integrant of Ex array described in 22519640 |
| OH15699 |  | *otIs716* | *str-97prom::GFP, rol-6(su1006)* | Integrant of Ex array described in (Vidal et al., 2018) |
| OH12495 |  | *otIs517* | *tph-1fosmid::SL2::YFP::H2B, ttx-3::mCherry* | (Serrano-Saiz et al., 2017) |
| SK4013 |  | *zdIs13* | *tph-1prom::GFP* | (Clark and Chiu, 2003) |
| OH15153 |  | *otIs661* | *unc-17prom::GFP, unc-122prom::GFP* | (Serrano-Saiz et al., 2020) |
| OH16393 |  | *otEx7503* | *unc-4prom::GFP::CLA-1, unc-4prom::tagRFP, rol-6(su1006)* | (Cook et al., 2020) |
| OH16337 |  | *otIs762* | *ceh-34prom::TagRFP, pha-1(+)* | This study |
| OH16700 |  | *otIs785* | *ceh-34prom::GFP::CLA-1, ceh-34prom::TagRFP, rol-6(su1006)* | This study |
| OH17856 |  | *otEx7922* | *ehs-1p::ttx-3, ehs-1p::unc-86, rol-6(su1006) – Line 1* | This study |
| OH17857 |  | *otEx7923* | *ehs-1p::ttx-3, ehs-1p::unc-86, rol-6(su1006) – Line 2* | This study |
| OH17858 |  | *otEx7924* | *ehs-1p::ttx-3, ehs-1p::unc-86, rol-6(su1006) – Line 3* | This study |

**References for strain list**

Amin, N.M., Lim, S.E., Shi, H., Chan, T.L., and Liu, J. (2009). A conserved Six-Eya cassette acts downstream of Wnt signaling to direct non-myogenic versus myogenic fates in the C. elegans postembryonic mesoderm. Dev Biol *331*, 350-360.

Aspock, G., Ruvkun, G., and Burglin, T.R. (2003). The Caenorhabditis elegans ems class homeobox gene ceh-2 is required for M3 pharynx motoneuron function. Development *130*, 3369-3378.

Baumeister, R., Liu, Y., and Ruvkun, G. (1996). Lineage-specific regulators couple cell lineage asymmetry to the transcription of the Caenorhabditis elegans POU gene unc-86 during neurogenesis. Genes Dev *10*, 1395-1410.

Bayer, E., and Hobert, O. (2018). A novel null allele of C. elegans gene ceh-14. MicroPubl Biol *2018*.

Brockie, P.J., Madsen, D.M., Zheng, Y., Mellem, J., and Maricq, A.V. (2001). Differential expression of glutamate receptor subunits in the nervous system of Caenorhabditis elegans and their regulation by the homeodomain protein UNC-42. J Neurosci *21*, 1510-1522.

Clark, S.G., and Chiu, C. (2003). C. elegans ZAG-1, a Zn-finger-homeodomain protein, regulates axonal development and neuronal differentiation. Development *130*, 3781-3794.

Cook, S.J., Crouse, C.M., Yemini, E., Hall, D.H., Emmons, S.W., and Hobert, O. (2020). The connectome of the Caenorhabditis elegans pharynx. The Journal of comparative neurology.

Flames, N., and Hobert, O. (2009). Gene regulatory logic of dopamine neuron differentiation. Nature *458*, 885-889.

Furuya, M., Qadota, H., Chisholm, A.D., and Sugimoto, A. (2005). The C. elegans eyes absent ortholog EYA-1 is required for tissue differentiation and plays partially redundant roles with PAX-6. Dev Biol *286*, 452-463.

Hao, J.C., Yu, T.W., Fujisawa, K., Culotti, J.G., Gengyo-Ando, K., Mitani, S., Moulder, G., Barstead, R., Tessier-Lavigne, M., and Bargmann, C.I. (2001). C. elegans Slit Acts in Midline, Dorsal-Ventral, and Anterior-Posterior Guidance via the SAX-3/Robo Receptor. Neuron *32*, 25-38.

Hirose, T., Galvin, B.D., and Horvitz, H.R. (2010). Six and Eya promote apoptosis through direct transcriptional activation of the proapoptotic BH3-only gene egl-1 in Caenorhabditis elegans. Proc Natl Acad Sci U S A *107*, 15479-15484.

Kim, K., and Li, C. (2004). Expression and regulation of an FMRFamide-related neuropeptide gene family in Caenorhabditis elegans. The Journal of comparative neurology *475*, 540-550.

Leyva-Diaz, E., Stefanakis, N., Carrera, I., Glenwinkel, L., Wang, G., Driscoll, M., and Hobert, O. (2017). Silencing of Repetitive DNA Is Controlled by a Member of an Unusual Caenorhabditis elegans Gene Family. Genetics *207*, 529-545.

Liu, X., Long, F., Peng, H., Aerni, S.J., Jiang, M., Sanchez-Blanco, A., Murray, J.I., Preston, E., Mericle, B., Batzoglou, S.*, et al.* (2009). Analysis of cell fate from single-cell gene expression profiles in C. elegans. Cell *139*, 623-633.

Mango, S.E., Lambie, E.J., and Kimble, J. (1994). The pha-4 gene is required to generate the pharyngeal primordium of Caenorhabditis elegans. Development *120*, 3019-3031.

Nathoo, A.N., Moeller, R.A., Westlund, B.A., and Hart, A.C. (2001). Identification of neuropeptide-like protein gene families in Caenorhabditiselegans and other species. Proc Natl Acad Sci U S A *98*, 14000-14005.

Pereira, L., Aeschimann, F., Wang, C., Lawson, H., Serrano-Saiz, E., Portman, D.S., Grosshans, H., and Hobert, O. (2019). Timing mechanism of sexually dimorphic nervous system differentiation. eLife *8*.

Sando, S.R., Bhatla, N., Lee, E.L., and Horvitz, H.R. (2021). An hourglass circuit motif transforms a motor program via subcellularly localized muscle calcium signaling and contraction. eLife *10*.

Sarov, M., Murray, J.I., Schanze, K., Pozniakovski, A., Niu, W., Angermann, K., Hasse, S., Rupprecht, M., Vinis, E., Tinney, M.*, et al.* (2012). A genome-scale resource for in vivo tag-based protein function exploration in C. elegans. Cell *150*, 855-866.

Serrano-Saiz, E., Gulez, B., Pereira, L., Gendrel, M., Kerk, S.Y., Vidal, B., Feng, W., Wang, C., Kratsios, P., Rand, J.B.*, et al.* (2020). Modular Organization of Cis-regulatory Control Information of Neurotransmitter Pathway Genes in Caenorhabditis elegans. Genetics *215*, 665-681.

Serrano-Saiz, E., Pereira, L., Gendrel, M., Aghayeva, U., Battacharya, A., Howell, K., Garcia, L.R., and Hobert, O. (2017). A Neurotransmitter Atlas of the Caenorhabditis elegans Male Nervous System Reveals Sexually Dimorphic Neurotransmitter Usage. Genetics *206*, 1251-1269.

Serrano-Saiz, E., Poole, Richard J., Felton, T., Zhang, F., De La Cruz, Estanisla D., and Hobert, O. (2013). Modular Control of Glutamatergic Neuronal Identity in C. elegans by Distinct Homeodomain Proteins. Cell *155*, 659-673.

Stefanakis, N., Carrera, I., and Hobert, O. (2015). Regulatory Logic of Pan-Neuronal Gene Expression in C. elegans. Neuron *87*, 733-750.

Vidal, B., Aghayeva, U., Sun, H., Wang, C., Glenwinkel, L., Bayer, E.A., and Hobert, O. (2018). An atlas of Caenorhabditis elegans chemoreceptor expression. PLoS Biol *16*, e2004218.

Zhang, F., Bhattacharya, A., Nelson, J.C., Abe, N., Gordon, P., Lloret-Fernandez, C., Maicas, M., Flames, N., Mann, R.S., Colon-Ramos, D.A.*, et al.* (2014). The LIM and POU homeobox genes ttx-3 and unc-86 act as terminal selectors in distinct cholinergic and serotonergic neuron types. Development *141*, 422-435.

Zhang, L., Ward, J.D., Cheng, Z., and Dernburg, A.F. (2015). The auxin-inducible degradation (AID) system enables versatile conditional protein depletion in C. elegans. Development *142*, 4374-4384.
